# Tangential Flow Microfiltration for Viral Separation and Concentration

**DOI:** 10.3390/mi10050320

**Published:** 2019-05-12

**Authors:** Yi Wang, Keely Keller, Xuanhong Cheng

**Affiliations:** 1 Department of Materials Science and Engineering, Lehigh University, Bethlehem, PA 18015, USA; yiw716@lehigh.edu; 2 Department of Bioengineering, Lehigh University, Bethlehem, PA 18015, USA; keely.ann.keller@gmail.com

**Keywords:** HIV diagnostics, cross-flow filtration, microfluidic device, COMSOL, nanoporous membrane

## Abstract

Microfluidic devices that allow biological particle separation and concentration have found wide applications in medical diagnosis. Here we present a viral separation polydimethylsiloxane (PDMS) device that combines tangential flow microfiltration and affinity capture to enrich HIV virus in a single flow-through fashion. The set-up contains a filtration device and a tandem resistance channel. The filtration device consists of two parallel flow channels separated by a polycarbonate nanoporous membrane. The resistance channel, with dimensions design-guided by COMSOL simulation, controls flow permeation through the membrane in the filtration device. A flow-dependent viral capture efficiency is observed, which likely reflects the interplay of several processes, including specific binding of target virus, physical deposition of non-specific particles, and membrane cleaning by shear flow. At the optimal flow rate, nearly 100% of viral particles in the permeate are captured on the membrane with various input viral concentrations. With its easy operation and consistent performance, this microfluidic device provides a potential solution for HIV sample preparation in resource-limited settings.

## 1. Introduction

Diagnosis of viral infection such as HIV is still largely limited by resources in high-incidence areas such as sub-Saharan Africa. Microfluidic devices have been widely explored to tackle this challenge as they offer the potential of miniaturized, portable devices that are easy to operate and provide results faster than traditional viral-load or immunoassay tests [1,2,3]. In terms of measuring the HIV concentration in circulation, i.e., the viral load, challenges exist to separate and enrich viral particles from a complex fluid that contains many non-specific particles in the similar size range. For example, after primary HIV infection, the viral load could raise to thousands of particles per microliter of blood while there could easily be billions of extracellular vesicles in the same volume [4,5]. It has been an active research topic to efficiently extract viral particles of interest with simple procedures to facilitate subsequent viral detection.

Various microfluidic devices have been fabricated to separate, sort and concentrate biological particles from complex fluids [6,7,8]. Paper-based microfluidic devices have been introduced for point-of-care testing especially in resource-limited settings, as they are inexpensive to fabricate and allow passive transport of fluids without active pumping [9,10,11,12]. PDMS-based microfluidic devices, on the other hand, have improved optical, thermal and mechanical properties compared to paper, glass or silicon-based ‘lab-on-a-chip’ devices [1,13,14,15]. Depending on the mechanisms, the methods can be divided into physical and affinity separation approaches. Different physical properties, such as particle size, density, shape, electrical and dielectrical properties have been employed to separate viruses from other species. While they are flexible and have high throughputs, electrical separations often require special solutions to suspend the sample to reduce joule heating and/or electrical field shielding. Acoustic and optical separations also face the challenge of heating, especially in separating sub-micron and nanometer-sized species. Hydrodynamic separations, including pinched, inertia and dean flow fractionation are often not effective to separate nanoparticles. Dead-end filtration, although easy to operate, is prone to clogging. Furthermore, these physical methods generally lack specificity, as the target particles usually share similar physical properties to some of the non-specific species [6,8,16,17,18]. Affinity separation uses a solid matrix decorated with a receptor to specifically bind the target particles. For example, magnetic separation uses affinity magnetic beads combined with a magnetic field to temporarily immobilize the bound species while washing away all the other species. Although easy to operate and versatile towards targets of different sizes, magnetic separation could face variable yields especially at low target concentrations [19,20,21]. Flatbed microfluidic channels coated with antibodies have low capture efficiency for viruses due to the size mismatch between the channel and target particles. Introducing micro- and nanostructures such as posts and pores into microchannels or using nanofludics directly could significantly improve the interactions and capture yield, whereas they face issues such as fabrication complexity, high flow resistance and low throughput [22,23]. 

To address the challenges discussed above, especially the lack of specificity in physical separation and low throughput in affinity isolation of virus, we propose the combination of tangential flow microfiltration and affinity capture to separate HIV viruses. Nanofiltration is widely used in the pharmaceutical industry to eliminate virus contamination in blood-based products, using membranes with cut-off sizes smaller than viruses [24,25]. To improve throughput and reduce cake formation, one typical approach is cross-flow filtration, also referred to as tangential filtration. The fundamental idea is to force solute through a membrane while maintaining a tangential flow to clean the membrane. Compared to dead-end filtration, cross-flow filtration is able to achieve high separation efficiency while maintaining a high throughput by reducing membrane clogging. Beyond the conventional use to eliminate undesirable virus contamination, cross-flow filtration has been applied to harvest viruses and cells in microfluidic devices [26,27,28,29,30,31]. While cross-flow microfiltration separates species based on the physical size alone, functionalizing the membrane with affinity chemistry renders it specifically towards the surface biochemistry of the target species. Combining the tangential microfiltration and immunoaffinity capture methods in a microfluidic channel, Mittal et al. demonstrated isolation of rare cancer cells with a capture efficiency of 70% [32]. However, to our knowledge, the approach has not been evaluated for virus separation. 

**Figure 1:**
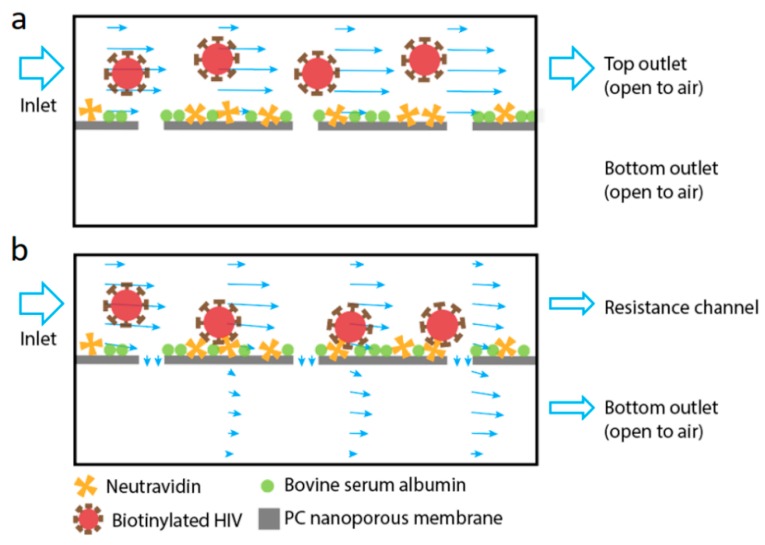
Schematics of the working principle of the device used in this work. The device is comprised of two flat channels separated vertically by a polycarbonate (PC) nanoporous membrane (pore size of 50 nm). The membrane is functionalized with NeutrAvidin to capture the biotinylated virus, with nonspecific binding blocked by bovine serum albumin. (**a**) When there is no cross-membrane flow, the virus primarily flows through the top channel. (**b**) When filtration is promoted using a resistance channel connected to the top channel, fluid enters the device through the top channel inlet and exits from both top (retentate) and bottom (permeate) outlets of the filtration device. The virus is pushed onto the membrane and captured by affinity binding. The arrows in the channels indicate local fluid velocity magnitude and direction.

In this work, we utilize a tangential flow polydimethylsiloxane (PDMS) device with a sandwiched nanoporous membrane to isolate and concentrate HIV virus. Cross-flow filtration is coupled with affinity separation to promote specificity and efficiency of HIV capture. As shown in Figure 1b, by applying an external resistance channel to control the permeation volume, the viral sample is partially pushed towards the membrane in the top channel and partially moves tangential to the membrane. Such diverted flow promotes the interaction between viral particles and the functional membrane surface. During filtration, particles smaller than the pores are able to cross the membrane and exit via the bottom channel outlet (permeate) while large particles in the sample exit through the top outlet (retentate). This easy-to-operate approach offers efficient viral sample processing with high throughput.

## 2. Materials and Methods

### 2.1. Materials

SU-8 photoresist was purchased from MicroChem (Newton, MA, USA). 3″ silicon wafers were purchased from Silicon Inc. (Boise, ID, USA). Sylgard 184 silicone elastomer kit was obtained from Dow Corning (Midland, MI, USA). Nuclepore track-etched polycarbonate (PC) membrane (pore size of 50 nm) was purchased from Thomas Scientific (Swedesboro, NJ, USA). Toluene and lyophilized bovine serum albumin (BSA) were obtained from Sigma-Aldrich (St. Louis, MO, USA). Phosphate buffered saline (PBS) was obtained from Mediatech (Herndon, VA, USA). NeutrAvidin and triton-X 100 were purchased from Thermo Scientific (Rockford, IL, USA). An HIV-1 p24 enzyme-linked immunosorbent assay (ELISA) kit was obtained from PerkinElmer (Waltham, MA, USA).

### 2.2. Assembly of Porous Membrane and Main Channels

**Figure 2:**
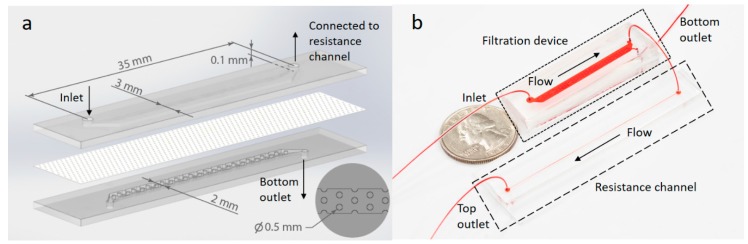
Schematic and photograph of the device. (**a**) A schematic showing the filtration device. A nanoporous PC membrane (50 nm pores) was assembled between two PDMS slabs containing molded microfluidic channels. The inlet in the top channel and outlets of both channels are labeled together with channel dimensions. The bottom right inset shows a top view of the molded PDMS posts which are regularly distributed in the bottom channel to physically support the membrane. (**b**) Photograph of the filtration device connected to a 65 mm long resistance channel. The devices are filled with food dye for visualization.

As shown in Figure 2, the filtration device contains a top channel and a bottom channel separated by a track-etched PC membrane with 50 nm pores. The height and length of two channels are identical of 0.1 mm and 30 mm while the top channel is 3 mm in width and 1 mm wider than the bottom channel for ease of channel alignment during device assembly. The bottom channel also contains cylinder posts 0.5 mm in diameter to support the membrane from sagging. Post positions are shown in the inset in Figure 2a.

Both the top and bottom PDMS channels were fabricated by standard soft lithography techniques. Briefly, SU-8 photoresist was patterned on silicon wafers in the clean room using standard photolithography. Afterwards, PDMS base and curing agent (Sylgard 184 silicone elastomer kit) were mixed at a 10:1 ratio and poured on the wafer molds. After 30-min degassing, the pre-polymer mixture was baked at 60 °C overnight to fully cure the PDMS. The cured PDMS was then removed from the molds, resulting in an open top channel and a post-containing bottom channel. The inlet and outlet ports of each channel were punched using a blunt-tip needle. 

Next, a 50-nm pore-size PC membrane with a surface area comparable to the PDMS slab/channel was adhered between the two channels using a PDMS ‘glue’ which is a mixture of PDMS pre-polymer and toluene in a 1:1 ratio (w/w) [33]. 0.5 mL of the glue mixture was spin-coated on a glass slide (25 mm × 75 mm) for 1 min at 2000 rpm. As soon as spinning was finished, PDMS slabs/channels were placed on the glass slide with the channel side facing down for 30 s. The PC membrane was placed on top of the glue-coated bottom channel, and then the glue-coated top channel PDMS layer was aligned and put on top of the membrane and bottom channel. The thinly spin-coated layer of glue prevented glue from squeezing into or occluding the PDMS channels during assembly. Assembled devices were baked at 60 °C for 5 h to fully cure the PDMS glue. Pieces of tubing (10 cm long) were inserted into the inlet and outlet ports.

### 2.3. Resistance Channel and Flow Tests

A thin and straight microchannel was connected with the top outlet of the filtration device to control the flow resistance in the tangential flow path. The resistance channel is 0.2 mm in width, 0.04 mm in height and 65 mm in length. The procedure to fabricate the resistance channel in PDMS using standard soft lithography is the same as that described above for the filtration channels. The cured PDMS resistance channel was bonded to a glass slide (75 mm × 25 mm) using oxygen plasma (Nordson MARCH, Concord, CA, USA). 

Flow tests were performed with and without the resistance channel connected to the top outlet (retentate) of the filtration device. 250 μL PBS was introduced into the top inlet of the filtration devices at different flow rates using a syringe pump (Chemyx, Stafford, TX, USA). Outflow was collected from both the retentate and permeate outlets, and weighed to calculate the volume ratio of solution draining through the membrane.

### 2.4. Membrane Functionalization and Viral Capture Test

Before the viral capture test, 250 μL deionized water was first injected by hand through the top inlet of the filtration device followed by 250 μL PBS buffer injection. Then, 150 μL NeutrAvidin at a concentration of 1 mg/mL in 1% BSA/PBS (w/v) was injected and incubated at room temperature for 2 h for membrane functionalization. Afterwards, PBS was hand injected to remove loosely bound NeutrAvidin from the channel. Next, the device was filled with 1% BSA/PBS solution and incubated at room temperature for 30 min in order to block any bare uncoated membrane. PBS buffer was used to wash the channel and purge air bubbles afterwards. At each incubation step, the tubing was clamped to avoid any bubbles being introduced into the device. No resistance channel was used in the membrane functionalization procedure. After membrane functionalization, the resistance channel was connected to the top outlet of the filtration device to add flow resistance to the tangential flow path. 

Pseudotyped HIV was cultured [34] and biotinylated [35] as reported before, and diluted to final concentrations using 1% BSA/PBS. 250 μL viral solution was pumped from the top inlet at defined flow rates. After this virus capture step, the resistance channel was disconnected and PBS was used to flush away unbound virus. Subsequently, 250 µL 0.5% Triton X-100 was injected at 2400 µL/h flow rate using a syringe pump from the top inlet to lyse the captured virus. Outflow from the top channel outlet was collected. HIV concentration in the lysate was determined using a commercial p24 ELISA assay according to the manufacturer’s recommended procedures. The HIV concentration in the lysate was compared to the input HIV concentration in order to calculate the virus capture efficiency.

### 2.5. Fluid Dynamics Simulation

3D simulation was carried out by COMSOL Multiphysics software (version 5.3a, COMSOL Inc., Stockholm, Sweden) to optimize fluid flow in the microfluidic channel with a sandwiched membrane. The simulated dimensions of the filtration device match those used experimentally. Stabilizing posts are not included in the simulation. Water was selected as the fluid in the channel domains. The membrane domain was set as a 10-µm-thick porous matrix with 10% porosity and permeability of 2.02 × 10^−18^ m^2^, which were obtained from the manufacturer’s data sheet and independent filtration tests, respectively. The flow in both the top and bottom channels was assumed to be steady state laminar flow and was described by the Navier–Stokes equation, while the membrane region was characterized by a Forchheimer-corrected version of the Brinkman equation [36]. A user-controlled octahedron mesh was applied to all the domains. Mesh convergence was confirmed. A no-slip boundary condition was applied to all solid walls. 

## 3. Results

Combining cross-flow microfiltration and affinity capture, we hypothesize that a high capture efficiency of viral particles can be achieved with continuous flow in a microfluidic channel. The filtration device consists of two parallel channels separated by a permeable PC nanoporous membrane. The viral sample flows in from the inlet at the top channel whereas two outlets are available at the other end of both the top and bottom channels. Thus, the sample can flow parallel to the membrane out of the top outlet as the retentate, or cross the membrane and exit from the bottom outlet as the permeate. The cross-membrane flow carries bioparticles from the top channel towards the membrane for their capture, while the tangential flow above the membrane continuously cleans the membrane from clogging. Specific viral capture is achieved through pre-functionalization of the membrane. Here, we study how the viral capture is influenced by top channel flow rates and viral concentrations.

### 3.1. Computational Analysis of Fluid Permeation through the Membrane

We first used COMSOL simulation to examine fluid flow in the proposed filtration device. To control the fraction of fluid exiting from the two channels separated by the membrane, an external straight microchannel is connected to the top retentate outlet of the filtration device, which increases the hydrodynamic resistance of the flow path tangential to the membrane. Without it, the membrane resistance is so high that little sample drains to the bottom permeate channel. As the resistance channel has a much smaller cross section compared to the top channel in the filtration device, it has much greater resistance, thus dominates the total resistance of the tangential flow path. In the membrane permeation flow path, the flow resistance is dominated by the porous membrane. Thus, by changing the length of the resistance channel, the permeation volume through the membrane can be readily controlled. 

**Figure 3:**
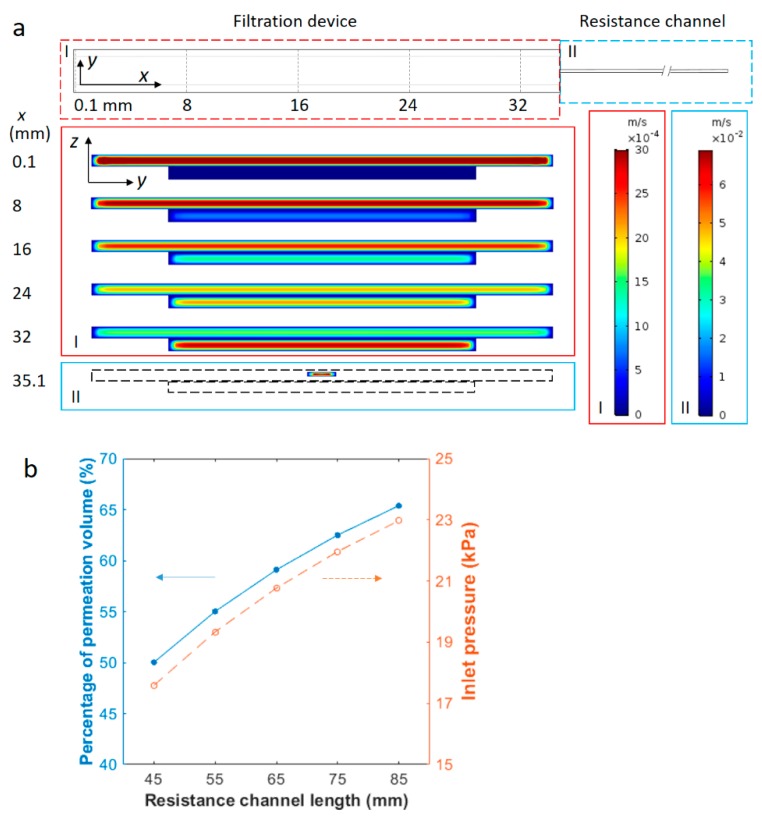
Magnitude profile of +*x* velocity and permeation percentage in the device from COMSOL simulation. (**a**) The top view of the simulated device and profiles of the axial velocity in *y*-*z* cross sections of the channel at different distances from the inlet. The simulated geometry contains a filtration device (region I) of two channels separated by a porous membrane and a 65-mm-long resistance channel (region II). The top channel of the filtration device is connected to the resistance channel, while the bottom channel is open to air at the outlet. The velocity profiles correspond to positions of different cross sections along the +*x* direction. These positions are labeled on the left of the velocity profiles in values (mm) and also as dashed lines in the *x*-*y* view of the simulated device (top). Axial flow is along the +*x* direction and the average inlet velocity is 2.4 mm/s. The left color legend of velocity magnitudes corresponds to the filtration device (region I) and right color legend corresponds to the resistance channel (region II). The magnitude of the axial velocity is observed to decrease in the top channel, but increase gradually in the bottom channel from the inlet to the outlet. (**b**) Fluid fraction permeating through the membrane and inlet pressure as a function of the resistance channel length. The blue solid circles correspond to the left *y*-axis and empty orange circles correspond to the right *y*-axis. The lines are to guide the eyes. The cross-sectional area of the resistance channel is constant. The average flow velocity at the inlet is 2.4 mm/s in all cases.

To guide the design of the resistance channel, the same dimension of the filtration device was used in the simulation as the experimental device (Figure 2a), and a permeable membrane separated the top and bottom channels. The resistance channel was simulated as an extension of the top channel beyond the filtration region (top of Figure 3a). It was 0.04 mm in depth and 0.2 mm in width, and with varying length from 45 to 85 mm. The two outlets, one at the bottom filtration channel and the other at the end of the resistance channel, were set to atmospheric pressure. The normal flow velocity at the inlet (at the left end of the top channel) was fixed at 2.4 mm/s in the simulation, which corresponded to a volumetric flow rate of 2400 μL/h.

An example of the simulation results is shown in Figure 3a. The resistance channel is 65 mm long in this case. Enlarged *y*-*z* cross sections in Figure 3a exhibit the *x*-component velocity profile at different locations from the inlet along the axial direction (+*x* direction). As demonstrated by the velocity profile in various cross sections normal to the axial flow, the fluid velocity gradually decreases in the top channel and increases in the bottom permeate channel. This indicates that the fluid from the inlet gradually drains through the porous membrane and flows out from outlets above and below the membrane. The volumetric flow rates, obtained by surface integration of the axial velocity, yield 980 µL/h and 1420 µL/h at the top and bottom outlets respectively, corresponding to a fraction of permeation of 59.10%. The inlet pressure is around 20.8 kPa from the simulation. The average axial velocity in the bottom channel exceeds that of the top channel towards the end of the filtration device due partially to the greater volumetric flow rate and partially to the smaller cross-sectional area of the bottom channel. When the permeation fraction was evaluated for different inlet velocities of 0.3, 1.0, 1.7 and 3.1 mm/s, the value was found to be consistently around 59.10% and independent of the sample flow rate. This is understandable, as the ratio of the volumetric flow rate in the two parallel flow paths is equal to the inverse ratio of the flow resistance, and is independent of the input flow rate. The inlet pressure was also evaluated from the COMSOL simulation and was found to increase linearly with the average input velocity, changing from 2.6 kPa at 0.3 mm/s to 26.8 kPa at 3.1 mm/s. All of these pressure values are well below the burst pressure of the sandwiched device, which was found to be 70–100 kPa using a homemade pressure-testing device. 

The fraction of permeated fluid volume was then simulated with various resistance channel lengths. Using the same cross-sectional area of 0.04 mm × 0.2 mm, channels of 45, 55, 65, 75 and 85 mm in length were tested at 2.4 mm/s inlet flow velocity. As the hydrodynamic resistance of a rectangular channel is proportional to its length, the longer resistance channels are expected to divert a larger amount of fluid to the bottom channel through the membrane. Results in Figure 3b show that the ratio of permeation volume rises from 50.02% with a 45 mm resistance channel to 65.38% with an 85 mm one. The inlet pressure increased from around 17.6 to 23.0 kPa, accordingly, due to greater flow resistance. The results indicate that the permeation volume can be easily controlled using properly selected resistance channels.

### 3.2. Experimental Evaluation of Fluid Permeation

Clogging is one of the main obstacles in microfiltration-based separation devices. Pressure accumulation caused by membrane fouling and clogging often lead to leakage and failure of the filtration device. In a tangential filtration device, however, the tangential flow clears the membrane fouling continuously, leading to greater permeate rate and extension of the device lifetime. As described above, the maximum backpressure and permeation fraction are controllable by the resistance channel design. In addition, sealing of the membrane in between the microchannels was also optimized to improve device integrity. To seal the membrane between two channels, we used a mortar layer of PDMS prepolymer as the glue between the PDMS slabs and the PC membrane [33]. A thin layer of PDMS precursor was spin-coated on the surface of a glass slide. The thickness of PDMS layer was controlled by the spinning speed and time [37]. Inked on the top and bottom PDMS blocks/channels, the precursor was able to penetrate through the porous membrane and hold on to the PDMS block/channel on the other side. After curing the glue layer at 60 °C, the membrane was sealed firmly between the two channels without any bubbles or membrane wrinkles. This method allows a uniformly thin layer of ‘glue’ to be transferred to the PDMS while accurately maintaining the channel geometry. Using a homemade pressure-testing device, the microfluidic device was found to be capable of holding up to 70–100 kPa of pressure. The maximum pressure in the experiments was designed to be less than 30 kPa through proper resistance channel selection to ensure integrity of the filtration device. 

**Figure 4:**
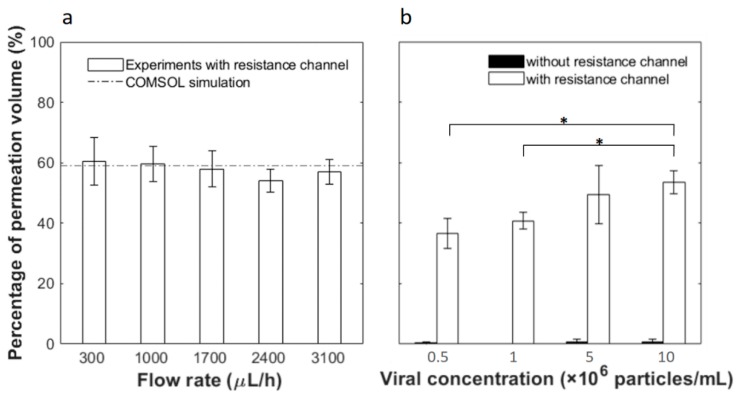
Fluid fraction permeating through the membrane as a function of (**a**) different flow rates and (**b**) inlet viral concentrations. In (**b**) the white bars are results from filtration devices connected with 65-mm-length resistance channels, while black bars are results from devices without. Error bars represent the standard deviation from at least 3 independent tests under the same condition. * indicates statistical difference based on two-tailed Student’s t-test at a 95% confidence interval.

Based on the analysis above, a 65-mm-length resistance channel was used to collect up to 60% filtrate. Flow tests were then carried out experimentally to examine the fluid permeation ratio at various flow rates and inlet viral concentrations. Permeation was first tested at different flow rates with PBS buffer and the results are shown in Figure 4a. As predicted, all fluid flows out of the top outlet without the resistance channel and no permeate is collectable from the bottom outlet (thus not plotted). With the 65 mm resistance channel, on the other hand, 60.54 ± 7.79%, 59.71 ± 5.78%, 57.95 ± 5.83%, 54.04 ± 3.82% and 56.94 ± 4.05% of the input fluid permeates through the membrane and flows out from the bottom outlet at respective flow rates of 300, 1000, 1700, 2400 and 3100 μL/h. No statistical difference is observed among the results at different flow rates. On average, the experimental permeation is 57.83 ± 2.56%, which compares well to the COMSOL simulation results of 59.10% permeation. 

Next, we flowed solutions containing pseudotyped HIV at various concentrations through the microfluidic device at the same inlet flow rate of 2400 μL/h. As shown in Figure 4b, without the resistance channel, outflow from the bottom outlet is less than 1% in all cases. With the resistance channel, the permeation volumes are 36.56 ± 4.86%, 40.75 ± 2.81%, 49.49 ± 9.68% and 53.54 ± 3.77% of the feed at respective input concentrations of 5 × 10^5^, 1 × 10^6^, 5 × 10^6^ and 1 × 10^7^ particles/mL. Thus, a greater input particle concentration yields greater fluid permeation. This is counter-intuitive as one would expect that more viral particles could potentially block more membrane pores, leading to less permeation. However, this phenomenon may be explained by the carrier solution used in this work. Since HIV was spiked in PBS containing 1% BSA and the membrane pore size is 50 nm, BSA aggregates could clog the pores. When a greater virus concentration was used, the sample would contain less carrier solution or reduced BSA interference, thus allowing greater permeation.

### 3.3. Viral Capture Efficiency 

**Figure 5:**
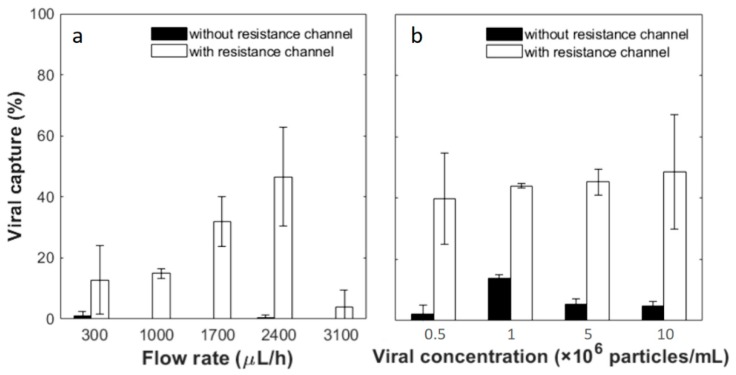
Viral capture yield as a function of (**a**) sample flow rates and (**b**) viral concentrations. Viral concentration used in (**a**) was 5 × 10^6^ particles/mL. The flow rate applied in (**b**) was 2400 µL/h. The white bars are results from filtration devices connected with the 65 mm resistance channel, while black bars are results from devices without. Error bars represent the standard deviation from at least 3 devices under the same conditions. Statistical analysis was conducted for (**b**) by applying an independent Student’s t-test at a 95% confidence interval and no statistically significant difference is observed among the white bars.

Viral capture tests were performed with biotinylated pseudotyped HIV in the tangential flow device with a pre-functionalized membrane. First, samples at a concentration of 5 × 10^6^ particles/mL were tested at different flow rates of 300, 1000, 1700, 2400 and 3100 μL/h. The resistance channel was disconnected from the device after applying the viral sample and the devices were rinsed with PBS at the same flow rate as the sample flow. Afterwards, Triton X-100 was flowed in to lyse the captured virus and release p24 antigen, the protein that makes up the core of HIV. Without the resistance channel, the lysis solution flowed almost exclusively out of the top outlet. Concentrations of p24 in the lysis solution and in the earlier flow-through solution were tested by a commercial ELISA assay. Mass conservation was confirmed by comparing the sum of the virus in different output fractions to that in the input. Capture efficiency was then calculated by the ratio of the p24 amount lysed out from the top channel to that in the input solution. As shown in Figure 5a, the capture efficiency is greatly dependent on the flow rate. A peak capture efficiency was found at flow rates of 1700–2400 μL/h. Viral capture percentage is 31.93 ± 8.25% at 1700 μL/h and 46.61 ± 16.19% at 2400 μL/h without statistical difference between these two flow conditions (95% confidence interval with Student’s t-test). At flow rates of 300, 1000 and 3100 μL/h, the capture efficiency further deceases to 4%–15%, and the difference in capture efficiency is significant between these flow rates and 2400 μL/h. Possible causes of this viral capture flow rate dependence are discussed later in the text. At each flow rate, control experiments were also carried out using filtration devices without resistance channels. In such cases, permeation through the membrane was negligible, as was the binding efficiency. The low capture efficiency without the resistance channel is understandable by comparing the characteristic time of different transport processes. The residence time of samples in the filtration microchannel is around 10–120 s at the flow rates tested, while the diffusion time of 100 nm diameter particles across a 0.05-mm-high channel is much greater, at about 250 s. Thus, when filtration is restricted, diffusion of viral particles to the channel walls is limited, resulting in low capture efficiency.

Next, a range of viral concentrations, 5 × 10^5^, 1 × 10^6^, 5 × 10^6^ and 1 × 10^7^ particles/mL, were tested as the input samples. The flow rate was fixed at 2400 μL/h, which showed the highest average yield at the concentration of 5 × 10^6^ particles/mL as described above. With the resistance channel, 39.68 ± 14.84%, 43.99 ± 0.67%, 45.21 ± 4.18% and 48.54 ± 18.70% of viral particles are captured from the input of the lowest to highest viral concentrations. The capture efficiency was further divided by the percentage of permeation volume to gain an understanding of capture from the filtered fraction. This yields capture efficiencies of 106.79 ± 26.39%, 108.60 ± 8.91%, 92.70 ± 11.40% and 90.51 ± 18.70%, respectively, in the permeated fraction, and there is no statistical difference among the different concentration groups (95% confidence interval with Student’s t-test). Without the resistance channel, the capture yields are significantly lower. From the input samples of the lowest to highest viral concentrations, 2.33 ± 2.71%, 13.95 ± 1.10%, 5.35 ± 1.80% and 4.68 ± 1.54% viral particles are captured. As little flow drained through the membrane without an external resistance channel according to COMSOL simulations and flow experiments discussed above, these yields indicate viral separation achieved by affinity capture only. This may explain capture efficiencies higher than 100% with resistance channels, after captures were normalized to permeation volume. Significant differences are observed between each pair of tests with and without the resistance channel at the same viral concentration. The results confirm that the tandem filtration device and resistance channel yield consistent and close to 100% viral capture from the membrane permeate in a wide range of input concentrations. 

## 4. Discussion

Overall, we implemented tangential flow filtration in microfluidic devices for viral capture in a high throughput and simple flow-through fashion. The device contains a filtration device coupled with a 65-mm-long resistance channel to achieve a 6:4 split in volume of the permeate versus retentate. While maintaining laminar flows in both the permeate and retentate compartments, the filtration process promotes viral interactions with the functionalized capture membrane, while the tangential flow continuously clears non-specific deposits on the membrane. Compared to the filtration device alone without the resistance channel, which could represent a flatbed capture channel (as no fluid permeates through the membrane), the tangential filtration device allows significantly improved capture yields. Flow rate-dependent yields were observed with a peak at 1700–2400 μL/h. Reduced capture at slow flow is likely a result of competition between specific capture and non-specific deposit on the membrane. Similar competition has been reported before for immunoaffinity cell capture from blood. For example, capture of CD4+ T lymphocytes from blood in a flatbed microfluidic channel is more efficient when the near-wall shear is strong enough to clear the surface of red blood cells [38]. In our work, although a cell-free sample of unpurified virus culture spiked in a BSA solution is used, the presence of extracellular vesicles and protein aggregates in the virus culture and carrier solution are also capable of interacting nonspecifically with the membrane, especially under the permeation pressures applied. According to Altmann et al., the deposition of a streaming particle in a crossflow microfluidic system depends on the balance of the lift force and drag force acting on the particle [39]. These hydrodynamic forces are functions of the particle size and flow rate. At low flow rates, all particles have a great chance to interact with the membrane, and nonspecific deposit in this case competes with specific capture of viral particles. On the other hand, high flow rates create great wall shear stress and short sample residence time, both of which lead to reduced binding of both specific and nonspecific species [32]. Thus, the peak capture efficiency at the flow rates 1700–2400 μL/h represents a balance where nonspecific binding is reduced by tangential flow, while specific binding is not yet reduced by the shear stress. 

In our tangential flow microfiltration device, membrane clogging is alleviated compared to dead end microfiltration, which often requires vibration or back flow periodically to disperse the cake layer [40,41,42]. Permeation is well controlled at flow rates up to 3100 μL/h and virus concentrations up to 1 × 10^7^ particles/mL. Combined with affinity capture, close to 100% virus is consistently captured on the membrane from the permeate. Compared to other viral separation devices reported in the literature, the devices described here have the advantages of greater capture yield, high operable flow rates and simple operation procedures [43,44,45]. 

Although biotinylated HIV particles and a NeutrAvidin coating are used in this work as proof of principle, the tangential filtration device is adaptable to other affinity chemistries targeting viral surface proteins. As permeation is easily controlled by the geometry of the resistance channel, the capture yield from the input stream is tunable and can be improved by either increasing the permeate fraction or feeding the retentate into the device repeatedly. As the target viral particles are captured through an affinity chemistry on a membrane within a microfluidic channel, enrichment and purification are both achieved. With recent development of optical and electrical bio-sensors [46,47], the filtration device could be developed into a diagnostic device by integrating sensors onto the capture membrane, an ongoing effort in the group. With simplified operation and fast sample processing speed, the device holds promise for viral analysis in resource-limited settings.

## 5. Conclusions

In conclusion, we successfully assembled a sandwich PDMS device incorporating a nanoporous PC membrane for viral particle separation. Combining tangential filtration and affinity capture, separation of HIV virus is demonstrated in continuous flow. The efficiency of viral capture is flow rate-dependent. At the optimal flow rate, close to 100% of viral particles are captured on the filtration membrane from the permeate with various viral concentrations. This low-cost and easy-to-operate device provides a promising solution to viral sample processing in resource-limited settings.
